# Supplementary material for: Neurocomputational mechanisms of biased impression formation in lonely individuals
Source: Commun Biol. 2023 Nov 3;6:1118. doi: 10.1038/s42003-023-05429-2 (PMC10624906; doi:10.1038/s42003-023-05429-2)
Supplement: Supplementary file 2 — Supplementary Material [file 42003_2023_5429_MOESM2_ESM.pdf]

Fig. S1

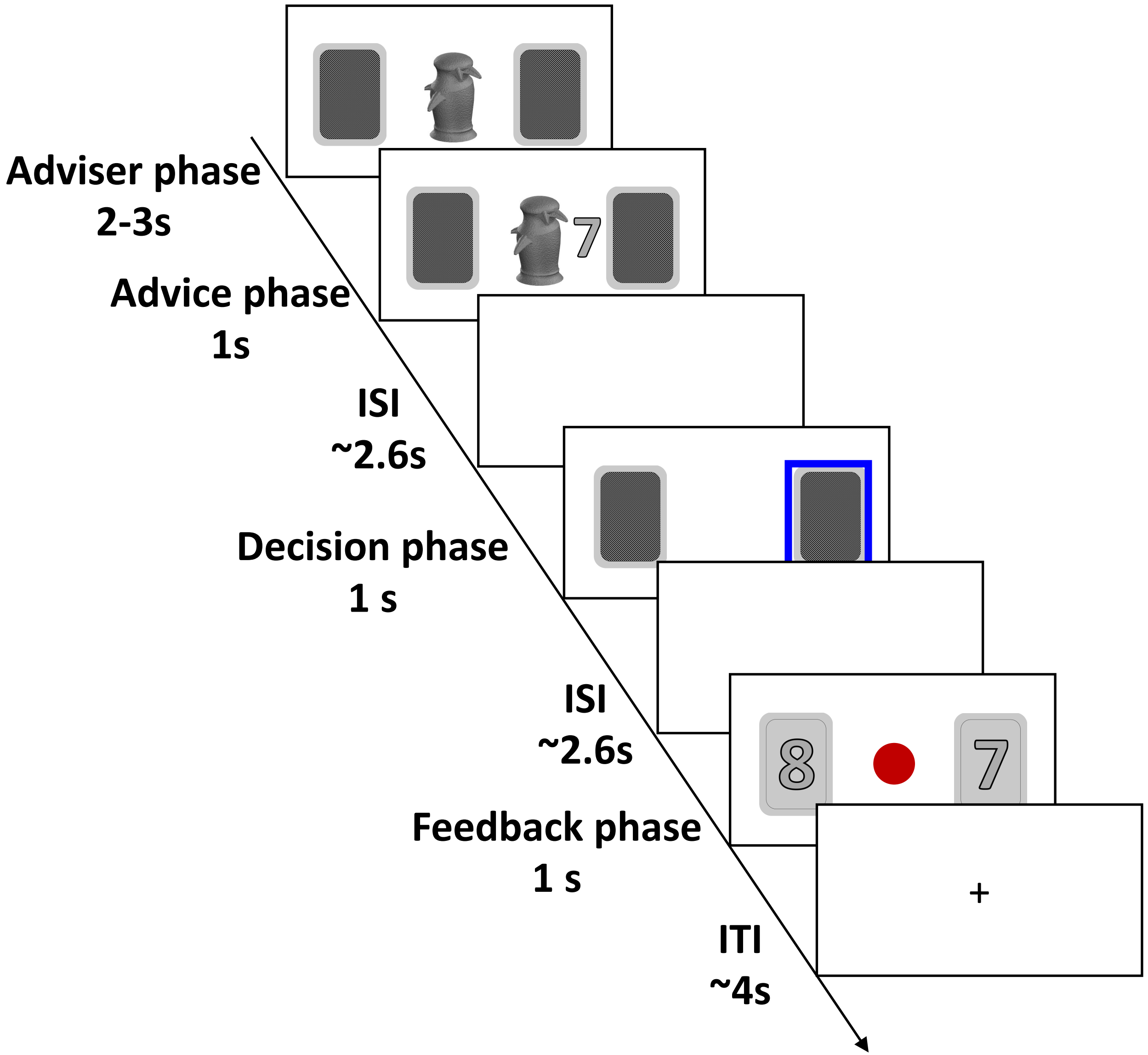

Adviser phase
2-3s
7
Advice phase
1s
ISI
~2.6s
Decision phase
1 s
ISI
~2.6s
8
7
Feedback phase
1 s
+
ITI
~4s

Fig. S2

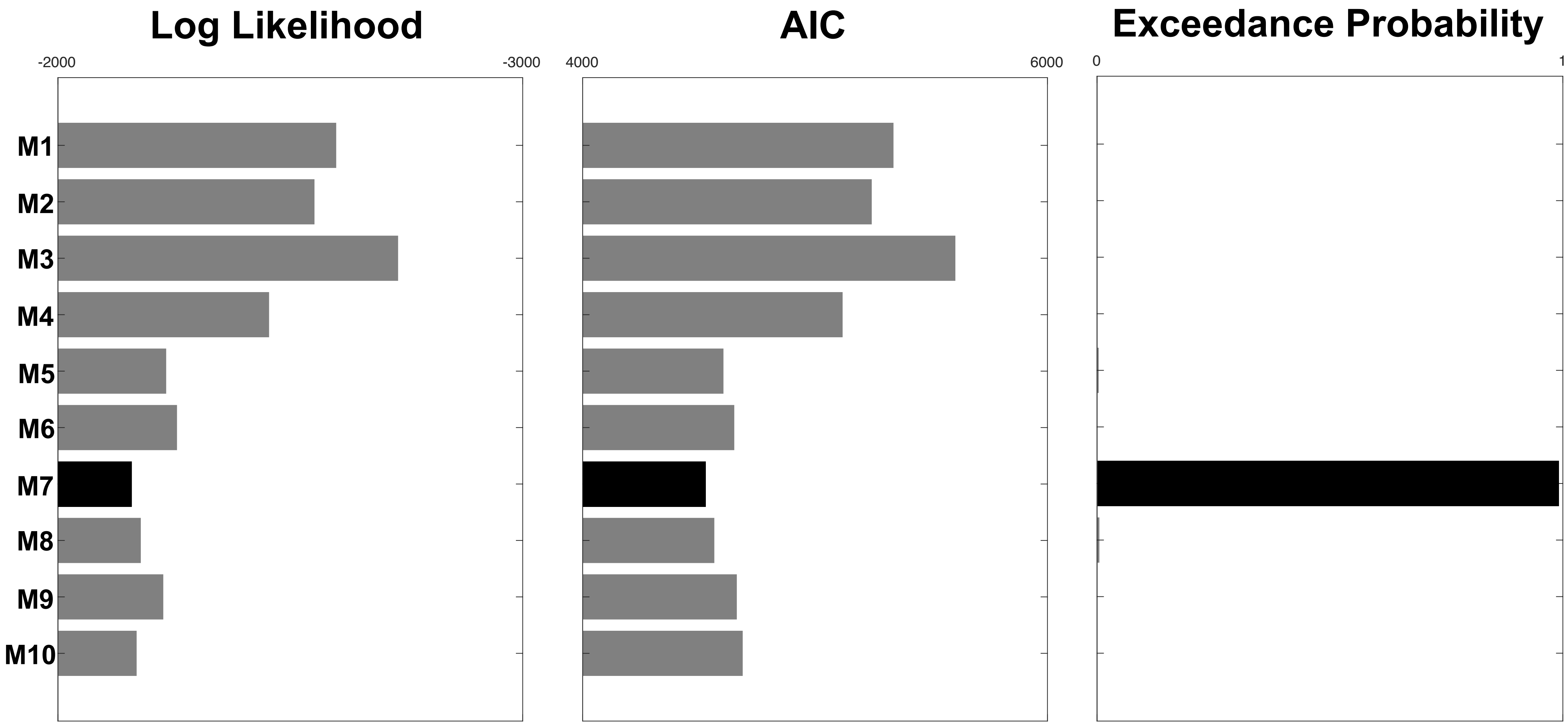
Log Likelihood
-2000
-3000
AIC
4000
6000
Exceedance Probability
0
1
M1
M2
M3
M4
M5
M6
M7
M8
M9
M10

Captions to Supplementary Figures

Fig. S1. Paradigm. Timeline of the Take Advice Game (TAG) in the MRI scanner. The task consisted of 4 phases. In the adviser phase, participants were presented with the avatar of the the other co-player (adviser) who would advise them in that trial. After a brief random delay (2-3 seconds after adviser presentation) the advice was presented (presentation time: 1s). The advice was a number between 1 and 9 (expect for 5) either next to the right or the left card. After a variable ISI (range: 2-8s, mean: 2.6s), participants needed to choose one of the two cards (1s). After another variable ISI, in the feedback phase (1s), participants saw the card numbers based on which they could judge the honesty of the adviser (social information), and a red or green circle between the cards, representing the participant's performance (nonsocial information). Between trials, an ITI (range: 2-8s, mean: 4s) showing a fixation cross was presented. ISI, interstimulus interval; ITI, intertrial interval.

Fig. S2. Random-effects Bayesian model comparison. Log likelihood, Akaike Information Criterion (AIC) and exceedance probability for the ten models of the model space considered. The winning model is represented by the black bar. Overall, better models have higher log likelihood, lower AIC and higher exceedance probability.

Supplemental Tables

Table S1. Neural correlates of surprise signals.

Hemisphere	Brain Region	Anatomical label	BA (PCM)	MNI Coordinates (mm)			*k*	T
				x	y	z		
**Positive**								
**L**	**Fusiform area**	**fusiform gyrus**	**37 (FG3)**	**-28**	**-48**	**-14**	**7694**	**8.48**
	hippocampus/amygdala		20 (CA1-2/AStr)	-26	-10	-18		7.11
	TPJ	superior temporal gyrus	42 (PFop/PFcm)	-58	-30	22		5.52
**R**	**Fusiform area**	**fusiform gyrus**	**37 (FG3)**	**32**	**-42**	**-10**	**1580**	**5.23**
	fusiform area	fusiform gyrus	37 (FG3)	34	-48	-4		5.11
	hippocampus	subiculum	30	24	-32	-16		4.54
**R**	**TPJ**	**supramarginal gyrus**	**42 (OP1/PFop/PFcm)**	**56**	**-24**	**18**	**4230**	**5.15**
	TPJ	supramarginal gyrus	42 (PFcm/PF)	58	-30	28		4.93
	TPJ	superior temporal gyrus	42 (PFcm/OP1)	52	-34	18		4.90
**L**	**OFC**	**middle orbital frontal gyrus**	**11 (s32/s24)**	**-6**	**34**	**-12**	**706**	**4.83**
	dACC		24	-2	34	14		4.48
R	OFC	middle orbital frontal gyrus	11 (s32)	6	40	-12		4.24
**L**	**DLPFC**	**superior frontal gyrus**	**9**	**-24**	**30**	**34**	**334**	**4.81**

	DLPFC	middle frontal gyrus	9	-28	36	26		4.13
	DLPFC	middle frontal gyrus	9	-26	34	46		3.95
**L**	**Cerebellum**		**(VIII)**	**-22**	**-58**	**-54**		**4.42**
	cerebellum		(VIII)	-34	-52	-50		4.21
	cerebellum		(VIII)	-16	-66	-50		3.83
**Negative**								
**R**	**DLPFC**	**middle frontal gyrus**	**44**	**40**	**12**	**38**	**838**	**5.48**
	DLPFC	inferior frontal gyrus (p. triangularis)	45	50	24	16		4.51
	DLPFC	inferior frontal gyrus (p. triangularis)	45	54	30	20		4.24
**R**	**FEF**	**superior medial gyrus**	**8**	**6**	**34**	**50**	**278**	**5.11**
**L**	**PMC**	**precentral gyrus**	**6**	**-40**	**4**	**36**	**536**	**4.51**
	DLPFC	inferior frontal gyrus (p. triangularis)	44	-44	14	28		3.76
	DLPFC	inferior frontal gyrus (p. triangularis)	45	-50	24	30		3.37
**R**	**IPL**		**40 (hlP3)**	**38**	**-52**	**48**	**228**	**4.38**
	angular gyrus		40 (hlP1/PGa/PFm)	46	-48	36		3.76
	IPL		40 (hlP2/hlP1/PFm)	48	-44	46		3.58

In bold is the brain region corresponding to the peak of the significant cluster. The other are brain regions of further peaks within the cluster. TPJ, temporoparietal junction; OFC, orbitofrontal cortex; dACC, dorsal anterior cingulate cortex; DLPFC, dorsolateral prefrontal cortex; FEF, frontal eye field; PMC, premotor cortex; IPL, inferior parietal lobule; R, right; L, left; BA, Broadman Area; PCM, probabilistic cytoarchitectonic map based on the SPM Anatomy toolbox.

Table S2. Neural correlates of the negativity bias

Hemisphere	Brain Region	Anatomical label	BA (PCM)	MNI Coordinates (mm)			*k*	T
				x	y	z		
**R**	**OFC/ACC**		**11**	**12**	**34**	**10**	**891**	**3.98**
L			11 (s32)	-14	42	-6		3.95
R	ACC		32/24	2	44	24		3.90
**R**	**caudate**			**8**	**2**	**10**	**768**	**4.65**
L	caudate			-10	12	14		3.58
L	caudate			-10	0	18		3.57

In bold is the brain region corresponding to the peak of the significant cluster. The other are brain regions of further peaks within the cluster. OFC, orbitofrontal cortex; ACC, anterior cingulate cortex; VTA, ventral tegmental area; TP, temporal pole; TC, temporal cortex; R, right; L, left; BA, Broadman Area; PCM, probabilistic cytoarchitectonic map based on the SPM Anatomy toolbox.
