# Supplementary material for: Neurocomputational mechanisms of biased impression formation in lonely individuals
Source: Commun Biol. 2023 Nov 3;6:1118. doi: 10.1038/s42003-023-05429-2 (PMC10624906; doi:10.1038/s42003-023-05429-2)
Supplement: Supplementary file 3 — Reporting Summary [file 42003_2023_5429_MOESM3_ESM.pdf]

nature portfolio

Corresponding author(s): DAPR

Last updated by author(s): May 18, 2023

Reporting Summary

Nature Portfolio wishes to improve the reproducibility of the work that we publish. This form provides structure for consistency and transparency in reporting. For further information on Nature Portfolio policies, see our Editorial Policies and the Editorial Policy Checklist.

Statistics

For all statistical analyses, confirm that the following items are present in the figure legend, table legend, main text, or Methods section.

n/a	Confirmed	
☐	☒	The exact sample size (*n*) for each experimental group/condition, given as a discrete number and unit of measurement
☒	☐	A statement on whether measurements were taken from distinct samples or whether the same sample was measured repeatedly
☐	☒	The statistical test(s) used AND whether they are one- or two-sided<br>*Only common tests should be described solely by name; describe more complex techniques in the Methods section.*
☐	☒	A description of all covariates tested
☐	☒	A description of any assumptions or corrections, such as tests of normality and adjustment for multiple comparisons
☐	☒	A full description of the statistical parameters including central tendency (e.g. means) or other basic estimates (e.g. regression coefficient) AND variation (e.g. standard deviation) or associated estimates of uncertainty (e.g. confidence intervals)
☐	☒	For null hypothesis testing, the test statistic (e.g. *F*, *t*, *r*) with confidence intervals, effect sizes, degrees of freedom and *P* value noted<br>*Give P values as exact values whenever suitable.*
☐	☒	For Bayesian analysis, information on the choice of priors and Markov chain Monte Carlo settings
☒	☐	For hierarchical and complex designs, identification of the appropriate level for tests and full reporting of outcomes
☐	☒	Estimates of effect sizes (e.g. Cohen's *d*, Pearson's *r*), indicating how they were calculated

Our web collection on statistics for biologists contains articles on many of the points above.

Software and code

Policy information about availability of computer code

Data collection	Matlab and PsychToolbox
Data analysis	Matlab and R

For manuscripts utilizing custom algorithms or software that are central to the research but not yet described in published literature, software must be made available to editors and reviewers. We strongly encourage code deposition in a community repository (e.g. GitHub). See the Nature Portfolio guidelines for submitting code & software for further information.

Data

Policy information about availability of data

All manuscripts must include a data availability statement. This statement should provide the following information, where applicable:

- Accession codes, unique identifiers, or web links for publicly available datasets
- A description of any restrictions on data availability
- For clinical datasets or third party data, please ensure that the statement adheres to our policy

Behavioral data and codes are accessible on OSF: https://osf.io/raf5h. Unthresholded maps of neural results are available at https://neurovault.org/collections/15181/.

Research involving human participants, their data, or biological material

Policy information about studies with human participants or human data. See also policy information about sex, gender (identity/presentation), and sexual orientation and race, ethnicity and racism.

Reporting on sex and gender	For descriptive purposes, we reported self-reported gender of our sample
Reporting on race, ethnicity, or other socially relevant groupings	Doesn't apply
Population characteristics	Participants' age was reported and that they were right-handed and had no history of mental disorders (assessed for MRI screening)
Recruitment	Participants were recruited from the student community at the University
Ethics oversight	Ethics committe at the University of Lübeck

Note that full information on the approval of the study protocol must also be provided in the manuscript.

Field-specific reporting

Please select the one below that is the best fit for your research. If you are not sure, read the appropriate sections before making your selection.

☒ Life sciences ☐ Behavioural & social sciences ☐ Ecological, evolutionary & environmental sciences

For a reference copy of the document with all sections, see nature.com/documents/nr-reporting-summary-flat.pdf

Life sciences study design

All studies must disclose on these points even when the disclosure is negative.

Sample size	Sample size was based on previous studies.
Data exclusions	Data of two participants were excluded due to technical issues.
Replication	Permutations, model comparisons and standard corrections for multiple comparisons were applied where appropriate to verify the robustness of our results.
Randomization	The study had a within-subject design and stimulus presentation was randomized within participants.
Blinding	Doesn't apply

Reporting for specific materials, systems and methods

We require information from authors about some types of materials, experimental systems and methods used in many studies. Here, indicate whether each material, system or method listed is relevant to your study. If you are not sure if a list item applies to your research, read the appropriate section before selecting a response.

Materials & experimental systems

n/a	Involved in the study	
☒	☐	Antibodies
☒	☐	Eukaryotic cell lines
☒	☐	Palaeontology and archaeology
☒	☐	Animals and other organisms
☒	☐	Clinical data
☒	☐	Dual use research of concern
☒	☐	Plants

Methods

n/a	Involved in the study	
☒	☐	ChIP-seq
☒	☐	Flow cytometry
☐	☒	MRI-based neuroimaging

Magnetic resonance imaging

Experimental design

Design type	Event-related block design with social learning task
Design specifications	Intertrial stimulus intervals (ISIs) were 2-8 (mean: 2.6s) seconds long. Jitters between trials were 2-8 (mean: 4s) seconds long. Participants played a total of 4 runs in the scanner with 66 trials each for a total of 264 trials.
Behavioral performance measures	Correct responses

Acquisition

Imaging type(s)	Structural and functional imaging
Field strength	3 T
Sequence & imaging parameters	The fMRI scans consisted of approximately 900 contiguous volumes per run (axial slices, 56; slice thickness, 3 mm; no interslice gap; TR, 1000 ms; TE, 30 ms; acceleration factor, 4; flip angle, 60°; voxel size, 3.0×3.0×3.0 mm3; FOV, 204×204 mm2). High-resolution structural images were acquired through a 3D sagittal T1-weighted MP-RAGE (sagittal slices, 208; TR, 2300 ms; TE, 2.43 ms; slice thickness, 0.85 mm; voxel size, .85×.85×.85 mm3; flip angle, 8°; inversion time, 1100 ms; FOV, 240×240 mm2).
Area of acquisition	Whole-brain scan.

Diffusion MRI ☐ Used ☒ Not used

Preprocessing

Preprocessing software	Neuroimaging data analyses were performed on SPM12 (v. 6905; http://www.fil.ion.ucl.ac.uk/spm/software/spm12/) in MATLAB 2019a (The Mathworks, Natick, Massachusetts; http://www.mathworks.com/). The functional images were corrected for slice acquisition time and voxel displacement using field maps, realigned for head movement correction to the mean image, co-registered to their structural images using the unified segmentation procedure (55), normalized into MNI space using deformation fields from the segmentation procedure (resampling voxel size: 2×2×2 mm3), and spatially smoothed using a Gaussian filter (8×8×8 mm3 full width at half maximum, FWHM) to decrease spatial noise
Normalization	Normalization into MNI space using deformation fields from the segmentation procedure.
Normalization template	MNI template provided by SPM
Noise and artifact removal	Motion parameters were control for as regressor of no interest in the GLMs
Volume censoring	Doesn't apply

Statistical modeling & inference

Model type and settings

We used both mass univariate and multivariate analyses.

For mass univariate analyses: on the first level, a general linear model (GLM) was estimated for each run with parametric modulators of the feedback phase containing model-based, trial-by-trial surprise estimates. For each task phase, two regressors were estimated (one for each adviser). For the feedback phase, a parametric modulator entailing trialwise surprise values was added. Moreover, motion parameters were included as regressors of no-interest. A temporal high-pass filter with a cutoff of 128 s was applied. Contrast analyses between beta regressors were performed on the first level and later used for second-level whole-brain analyses. On the second level, to examine the neural signatures of social surprise, a one-sample t-test on the parametric modulators of the feedback phase was performed at the second (group) level. To investigate the relationships between neural responses to social information and the impression-induced negativity bias, whole-brain contrast images of the feedback regressor were correlated with learning rate differences in a subject-level whole-brain regression analysis. To test the relationships between neural responses to social information and subjective feelings of loneliness, the same whole-brain contrast images of the feedback regressor were correlated with loneliness scores from the UCLA loneliness scale in a subject-level whole-brain regression analysis.
Results were whole-brain corrected for multiple comparisons using a voxel-level threshold of $p < .001$ and a family-wise error, cluster-level (FWEc) corrected threshold of $p < .05$. Small-volume correction for the OFC was based on an independent anatomical OFC volume provided by the SPM Anatomy toolbox (v. 2.2).

Multivariate analyses: to investigate the relationships of the neural signal in the OFC with participants' impressions of the advisers and their trustworthiness judgments, multivariate classification and regression analyses were performed. Between-adviser differences of OFC neural responses to social information were used as feature. Voxelwise betas were extracted from within the cluster yielded by the previous whole-brain regression analysis with the impression-induced negativity bias. Overall impression of an adviser as honest or dishonest was used as binary target for the classification analysis. The difference in trustworthiness ratings between advisers was used as continuous target for the regression analysis.
For the multivariate classification analysis, logistic boosting regression was employed building an ensemble of 500

classification trees with a learning rate parameter = .01 as implemented in fitcensemble in MATLAB with LogitBoot as method. For the multivariate regression analysis, least-square boosting regression was employed building an ensemble of 500 classification trees with a learning rate parameter = .01 as implemented in fitrensemble in MATLAB with LSBoost as method. For both multivariate analyses, a 20% left-out cross-validation approach was used where the algorithm was trained on 80% of the data and tested on the left-out 20% in each of the five folds. Cross-validated performance was tested against a permutation test with 10,000 permutations.

Effect(s) tested: We used a combination of classic statistical tests (e.g., t-test, mediation analyses), computational modeling and machine learning.

Specify type of analysis: ☐ Whole brain ☐ ROI-based ☒ Both

Anatomical location(s): OFC ROI was determined based on two different analyses on the second level that showed converging evidence of the involvement of the area.

Statistic type for inference (See Eklund et al. 2016): Results were reported at a voxel-wise threshold of p<.001 and a cluster-wise threshold of FWE<.05

Correction: FWE and permutations

Models & analysis

n/a	Involved in the study
☐	☒ Functional and/or effective connectivity
☒	☐ Graph analysis
☐	☒ Multivariate modeling or predictive analysis

Functional and/or effective connectivity: Psychophysiological interaction analysis

Multivariate modeling and predictive analysis: Logistic and least-square boosting regressions.
